# Burnout and posttraumatic stress among nurses in German care homes: a cross-sectional survey during the COVID-19 pandemic

**DOI:** 10.1186/s12912-026-05092-y

**Published:** 2026-07-21

**Authors:** Carolin Swoboda, Raphael Kohl, Adelheid Kuhlmey, Julie L. O‘Sullivan, Paul Gellert, Christian Hering

**Affiliations:** 1 Charité – Universitätsmedizin Berlin, Corporate Member of Freie Universität Berlin and Humboldt-Universität zu Berlin, Institute of Medical Sociology and Rehabilitation Science, Charitéplatz 1, 10117 Berlin, Germany; 2 Einstein Center Population Diversity (ECPD), Berlin, Germany; 3 German Center for Mental Health (DZPG), Partner Site Berlin-Potsdam, Berlin, Germany; 4 Friede Springer - Cardiovascular Prevention Center, Charité – Universitätsmedizin Berlin, Corporate Member of Freie Universität Berlin and Humboldt-Universität zu Berlin, Berlin, Germany

**Keywords:** PTSD, Long-term care, Nursing homes, Psychosocial support, COVID-19

## Abstract

**Background:**

The COVID-19 pandemic has imposed unprecedented psychological demands on nurses working in care homes, profoundly affecting their well-being. Thus, this cross-sectional study examined the psychological burden among German care home nurses during the fourth COVID-19 wave and the early phase of the fifth wave in Germany, focusing on posttraumatic stress disorder (PTSD) and burnout, and assessed the availability of workplace psychosocial resources.

**Methods:**

A total of 719 nurses completed an online cross-sectional convenience survey between October 2021 and January 2022. Given the voluntary online recruitment, the findings should be interpreted with caution due to the potential for self-selection bias. PTSD symptoms were assessed using the International Trauma Questionnaire (ITQ), and burnout dimensions were assessed using the Copenhagen Burnout Inventory (CBI). Multiple linear regression analyses were conducted to identify factors associated with PTSD and different dimensions of burnout, including personal burnout, work-related burnout and resident-related burnout.

**Results:**

Among nurses, 91.4% fulfilled PTSD screening criteria and/or exceeded the indicative CBI cut-off for at least one burnout dimension, indicating a high psychological burden within the present study sample. Specifically, 29.5% met screening criteria for PTSD, 90.0% exceeded the indicative CBI cut-off for personal burnout, 78.9% for work-related burnout, and 33.4% for resident-related burnout. Only 21.7% reported availability of psychosocial support at work. PTSD was significantly associated with poorer general health, lower job satisfaction, COVID-19-related deaths among residents in care home, dementia-focused care and female gender. Personal burnout was significantly associated with poorer general health, lower job satisfaction, female gender, absence of participatory leadership at care home, younger age, no COVID-19 infection history, and lower posttraumatic growth. Work-related burnout was significantly associated with lower job satisfaction, poorer general health, lower posttraumatic growth, female gender, no COVID-19 infection history, absence of participatory leadership at care home, and full-time employment. Resident-related burnout was significantly associated with lower job satisfaction, poorer general health, lower posttraumatic growth, and no COVID-19 infection history.

**Conclusion:**

The high levels of PTSD and burnout found in the present study likely reflect strain placed by the pandemic as well as constant work-related strain on care home nurses. These findings underscore the urgent need for improved psychosocial support and organizational strategies to strengthen mental health in long-term care settings.

**Clinical trial number:**

Not applicable.

**Supplementary Information:**

The online version contains supplementary material available at 10.1186/s12912-026-05092-y.

## Background

The COVID-19 pandemic posed major challenges for global health care systems, placing extraordinary physical and emotional demands on health care workers, including nurses working in care homes [1–3]. In Germany, these facilities were particularly affected during the second wave of the pandemic, which was marked by high mortality rates among residents and a substantial increase in staff workload as infection numbers surged [4]. Consequently, the strain on nurses working there was substantial [5, 6], placing them at elevated risk for adverse mental health outcomes, including burnout and posttraumatic stress disorder (PTSD). Meta-analytic studies report an overall burnout prevalence of 59.5% [7] and a pooled PTSD prevalence of 29.1% [8] among nurses during the COVID-19 pandemic. These rates exceed the general population and pre-pandemic levels, highlighting a significant mental health burden for nurses during the COVID-19 pandemic [9–11]. Several risk factors have been associated with burnout and PTSD in nurses and healthcare settings. Demographic and health-rated risk factors include female gender [12], younger age [9, 13], poor self-rated health [14], experiencing COVID-19 symptoms [15], a history of COVID-19 infections [16], and perceptions of COVID-19 as a threat and fear of infection [17, 18]. Occupational risk factors include full-time employment status [19, 20], understaffing [15], job dissatisfaction [14, 19, 21], not being able to participate in hospital affairs [22], working in healthcare settings with dementia-focused care [23, 24] and working in settings where patients died of COVID-19 [22]. Furthermore, PTSD and burnout have been shown to negatively affect quality and safety of patient care [25, 26]. In contrast, studies found that perceived organizational support [27], participation in work organization [28] and posttraumatic growth [29] have a protective function by being associated with lower levels of burnout or PTSD symptoms among nurses.

Theoretical frameworks such as the Job Demands–Resources (JD-R) model [30] and the Conservation of Resources (COR) theory [31] offer perspectives for understanding the links of work demands and resources to burnout and trauma-related symptoms. The JD-R model posits that burnout arises when job demands exceed coping resources, while COR theory suggests that chronic stress depletes personal and external resources, increasing vulnerability to trauma-related outcomes [32]. During the COVID-19 pandemic, nurses experienced chronic exhaustion [6], faced particularly high job demands [33] and limited resources, by potentially being unable to replenish depleted internal and external resources [34], which may have contributed to elevated burnout and PTSD symptoms. These frameworks underscore the relevance of workplace resources as protective factors against such outcomes. Despite growing evidence on burnout and PTSD among nurses during the COVID-19 pandemic, research specifically examining these outcomes in German care homes, particularly following the early pandemic waves, remains scarce, with limited attention to associated workplace resources.

This study aims to investigate burnout and PTSD among nurses in German care homes during the fourth and the early phase of the fifth COVID-19 wave in Germany, to assess the availability of workplace resources such as access to psychosocial services, participatory leadership and professional development opportunities, and to identify factors associated with the psychological burden.

## Methods

### Design and participants

An online survey among nurses working in German care homes was conducted between October 26th, 2021, and January 31st, 2022, as part of the *COVID-Heim* project. The project examined the impact of the COVID-19 pandemic on German care homes. Data collection of the present survey took place during the fourth COVID-19 wave in Germany and extended into the early phase of the fifth wave [35]. The survey comprised questions assessing current experiences (fourth and early fifth wave), as well as items referring to pandemic-related experiences since the pandemic’s onset (March 1, 2020). In addition, one retrospective item referred to the second wave of the pandemic (October 2020–January 2021) [36]. Eligible participants were nurses working in German care homes. Recruitment was carried out through multiple channels, including social media platforms and online forums, with a voluntary online convenience sampling strategy. Thus, the sample is non-probabilistic and may be affected by self-selection bias, which limits the representativeness of the findings for all nurses working in German care homes. Participants from all 16 German federal states were represented in the sample. Data was collected using a secure, web-based questionnaire administered via Research Electronic Data Capture (REDCap) platform [37]. The study was conducted in accordance with the Declaration of Helsinki. We followed the ‘Strengthening the Reporting of Observational Studies in Epidemiology’ (STROBE) checklist for cross-sectional studies. The survey of the present study is provided in Appendix A.

### Measurements

#### PTSD and burnout

PTSD and burnout, including personal, work-related, and resident-related burnout are the primary outcome measures of this study. PTSD was assessed using the International Trauma Questionnaire (ITQ), consisting of nine items rated on a five-point Likert scale (0 = “not at all” to 4 = “very strong”) evaluating traumatic symptoms over the past month [38]. In this study, participants rated all ITQ items with reference to their experiences during the COVID-19 pandemic and work-related stressful events, which served as the index event for the questionnaire. The ITQ measures symptoms of re-experiencing, avoidance, and perceived current threat, along with functional impairment, in line with the ICD-11 criteria for PTSD. According to the ITQ manual, a PTSD diagnosis requires the endorsement of at least one symptom from each cluster plus one indicator of functional impairment. In the present study, the ITQ was used to identify participants meeting PTSD screening criteria and to quantify PTSD symptom burden; it was not used to establish clinical PTSD diagnoses. For the present analyses, a dimensional PTSD score was calculated by summing the core symptom items, excluding those related to functional impairment [38], and used as a continuous outcome variable in the regression analyses. The ITQ demonstrated high internal consistency (Cronbach *α* = 0.90) in this study.

Burnout was measured using the 19-item Copenhagen Burnout Inventory (CBI), which assesses three dimensions of burnout, including personal burnout, work-related burnout, and client-related burnout [39]. Personal burnout captures general physical and emotional exhaustion regardless of work (e.g., “How often do you feel tired?”) and work-related burnout reflects exhaustion specifically related to work tasks (e.g., “Do you feel worn out at the end of the working day?”). For this study, the client-related subscale, which measures exhaustion resulting from direct contact with clients (e.g., “Does it drain your energy to work with clients?”), was adapted to focus on residents in the care homes (e.g., “Does it drain your energy to work with residents?”) and is therefore referred to as “resident-related burnout” hereafter. Each subscale comprises six to seven items rated on a five-point scale (0 = Never/Almost never, 25 = Rarely, 50 = Sometimes, 75 = Often, 100 = Always). Mean scores were calculated for analysis, with higher scores indicating greater exhaustion. A cut-off score of ≥ 50 was applied as an indicative threshold, also used in prior research [18, 40]. Scores exceeding the indicative CBI cut-off indicate elevated burnout symptoms but should not be interpreted as a clinical diagnosis of burnout. In this sample, the subscales demonstrated high internal consistency (Cronbach *α* = 0.91 for personal burnout, *α* = 0.90 for work-related burnout, and *α* = 0.90 for resident-related burnout).

#### Characteristics of participants and affiliated care homes

The online survey collected characteristics of participants by assessing sociodemographic data, including age, gender (female; male; divers) and employment status (e.g. full-time; part-time; marginal employment and other employment-types). Moreover, job satisfaction was assessed using a scale of the Copenhagen Psychosocial Questionnaire (COPSOQ), consisting of seven items [41]. Each item is rated on a five-point scale, with response weights ranging from 0 (very dissatisfied) to 100 (very satisfied) in increments of 25. Scores were aggregated to produce a mean value representing overall job satisfaction. The scale demonstrated high internal consistency (Cronbach *α* = 0.84) in this sample. Characteristics of affiliated care homes were assessed regarding the size of the care home (small: <50 residents; medium: 51–100; large: >100), availability of psychosocial services (“Does your institution offer psychosocial services [such as supervision, psychological counseling, etc.]?”), existence of participatory leadership in care home (“Does your institution provide nursing staff with the opportunity to actively participate in decision-making processes?”), and access to professional development opportunities (“Does your institution allow nursing staff to attend further training or continuing education courses?”). These items were captured dichotomously (Yes/No). Furthermore, the primary focus of care within the affiliated care home was assessed (e.g. dementia, mental illness, palliative care).

#### Health and posttraumatic growth

Health-related variables were assessed by capturing participants’ general health status, self-reported history of COVID-19 infection and posttraumatic growth. General health was assessed using a single item from COPSOQ [41]. Participants rated their current health on a 10-point rating scale (“Your state of health: If you evaluate the best conceivable state of health at 10 points and the worst at 0 points: How many points do you then give to your present state of health?”). COVID-19 infection history was determined via a dichotomous question (“Did you test positive for the coronavirus during the pandemic?”; Yes/No). Posttraumatic growth was measured using four items from the “Personal Strength” subscale of the Posttraumatic Growth Inventory (PTGI) [42] in the German validated version of the scale [43]. Items (e.g., “I have a greater feeling of self-reliance”) were then rated on a three-point Likert scale (0 = “not at all”, 1 = “somewhat”, 2 = “very much”) and summed to form a total score. Higher scores indicate greater posttraumatic growth. The PTGI subscale demonstrated high internal consistency (Cronbach *α* = 0.82) in this study.

#### COVID-19-related impact on care homes

COVID-19-related effects on nursing practice and care facilities were evaluated by assessing confirmed COVID-19 cases and deaths in care homes, staff absences and satisfaction with quality of resident care and support. Confirmed COVID-19 cases among staff and/or residents since the pandemic’s onset (March 1, 2020) were evaluated using binary (yes/no) items (“Since the beginning of the pandemic (March 1, 2020), have there been confirmed COVID-19 infections among nursing staff?” / “...among the residents?”). COVID-19-related deaths among residents since the onset of the pandemic were assessed with a binary (yes/no) item (“Since the beginning of the pandemic, have any residents in your facility died due to or in connection with a COVID-19 infection?”) as well. Perceived staff absences during the second wave were assessed via a targeted item: “Did staff absences occur during the second infection wave (October 1, 2020 to January 31, 2021)?”. Responses were recorded on a four-point Likert scale (0 = “strongly disagree”, 1 = “rather disagree”, 2 = “rather agree”, 3 = “strongly agree”). Satisfaction with quality of resident care and support was measured using a modified item [44], asking “How would you rate the overall quality of care and support for residents in your facility under the conditions of the COVID-19 pandemic?”. Responses utilized a four-point Likert scale (0 = “very dissatisfied”, 1 = “rather dissatisfied”, 2 = “rather satisfied”, 3 = “very satisfied”).

### Statistical analysis

Descriptive statistics were computed to analyze characteristics of participants and affiliated care homes. As the data was not normally distributed, non-parametric tests (Mann-Whitney U test) were applied to examine the relationship between PTSD, personal burnout, work-related burnout, resident-related burnout, and characteristics of participants and affiliated care homes. These Mann-Whitney U tests were conducted as exploratory analyses without formal adjustment for multiple testing; the corresponding results should therefore be interpreted as hypothesis-generating. For significant group differences, effect sizes (*r*) were calculated as: $\:r=\:\frac{\left|Z\right|}{\sqrt{N}}$ and reported accordingly. Predictor variables were selected based on theoretical considerations, previous literature on burnout and PTSD among healthcare workers, and their relevance to the study objectives. PTSD and burnout scores were analysed as continuous outcome variables; therefore, multiple linear regression was chosen to examine associations across the full range of symptom severity while preserving more information than dichotomised outcomes. The multiple linear regression models constituted the primary inferential analytical approach of this study. Multivariate associations between PTSD, burnout and characteristics of participants and affiliated care homes were examined using multiple linear regression models, which were fitted separately for PTSD, personal burnout, work-related burnout, and resident-related burnout. Regression assumptions were checked, and no violations or influential outliers were detected. Subsequently, all additional regression assumptions were examined, including the independence of residuals, absence of multicollinearity, homoscedasticity, and normal distribution of residuals. To assess these assumptions, the Durbin-Watson statistic, scatterplots, histograms, the variance inflation factor (VIF), and tolerance values were employed. These assumptions were found to be satisfied, as no high correlations (≥ 0.90) and no evidence of problematic multicollinearity were detected (all VIF values < 5; tolerance > 0.1). The observed VIF values and the Pearson correlation matrix are provided in Supplementary Tables S1 and S2, respectively. Outliers were assessed using studentized residuals, leverage values, and Cook’s distance, with no notable abnormalities identified. Only participants with complete data on the variables included in each respective analysis were retained; cases with missing values were excluded listwise. All analyses were performed using IBM SPSS Statistics version 30. Statistical significance was set at *p* < .05.

## Results

### Characteristics of participants and affiliated care homes

In the total sample of *N* = 719 nurses, most were female (87.3%, *n* = 628), with a mean age of 39.5 years (*SD* = 9.7) and employed full-time (67.3%, *n* = 484). Half of participating nurses worked in medium-sized care homes (51.3%, *n* = 369) and 22% (*n* = 158) of participants confirmed dementia-focused care at their care home. With regard to workplace resources, 21.7% (*n* = 156) reported having availability of psychosocial services in their care home, 32.7% (*n* = 235) reported the presence of participatory leadership at their care home, and 87.8% (*n* = 631) reported access to professional development opportunities. The mean job satisfaction in this sample was moderate (*M* = 45.43, *SD* = 19.92). Overall, 29.5% (*n* = 212) of participants met screening criteria for PTSD (ITQ). High levels of burnout were observed across dimensions (CBI), with 90.0% (*n* = 647, *M* = 70.64, *SD* = 17.83) of participants exceeding the indicative CBI cut-off for personal burnout, 78.9% (*n* = 567, *M* = 64.18, *SD* = 20.50) the cut-off for work-related burnout, and 33.4% (*n* = 240, *M* = 37.8, *SD* = 23.01) the cut-off for resident-related burnout. Taken together, 91.4% (*n* = 657) of participants met PTSD screening criteria and/or exceeded the indicative CBI cut-off for at least one burnout dimension, indicating elevated psychological symptom burden. Additional details on the distribution of CBI scores are presented in Supplementary Figure S3. The mean self-rated general health (COPSOQ) in the total sample was 5.15 (*SD* = 2.23) but appeared to be lower among nurses with any type of psychological burden. Approximately one quarter of the sample (24.5%, *n* = 176) reported a history of COVID-19 infection. Posttraumatic growth (PTGI) in the total sample was modest (*M* = 4.13, *SD* = 2.30) and tended to be lower in psychologically burdened nurses, reaching its lowest level among those with resident-related burnout (*M* = 3.51, *SD* = 2.19). All nurses reported that their care homes experienced COVID-19 cases among staff and/or residents (100%, *n* = 719), whereas 76% (*n* = 549) reported COVID-19-related deaths among residents in their care homes. Perceived staff absences during second wave were reported by 91.5% (*n* = 658) of participants. Satisfaction with quality of resident care and support was low, with only 32.1% (*n* = 231) of participants stating to be very / rather satisfied, while 68% (*n* = 488) of participants stated to be very / rather dissatisfied. Table 1 shows the characteristics of participants and affiliated care homes in detail.

**Table 1:** Characteristics of participants

	Total (*N* = 719)	PTSD (ITQ; *n* = 212)	Personal burnout (CBI; *n* = 647)	Work-related burnout (CBI; *n* = 567)	Resident-related burnout (CBI; *n* = 240)
Characteristics of participants and affiliated care homes					
Gender, *n* (%)					
Female	628 (87.3)	196 (96.5)	576 (89.0)	509 (89.8)	209 (87.1)
Male	89 (12.4)	16 (7.5)	69 (10.7)	57 (10.1)	30 (12.5)
Divers	2 (0.3)	-	2 (0.3)	1 (0.2)	1 (0.4)
Age (years), *M* (*SD*)	39.49 (9.72)	40.49 (9.94)	39.13 (9.61)	39.34 (9.70)	39.18 (10.0)
Employment status, *n* (%)					
Full-time	484 (67.3)	140 (66.0)	431 (66.6)	381 (67.2)	153 (63.7)
Part-time	227 (31.6)	70 (32.0)	208 (32.1)	178 (31.4)	83 (34.6)
Other ^a^	8 (1.2)	2 (0.9)	4 (0.6)	8 (1.4)	4 (1.7)
Size of care home, *n* (%)					
Small (1–50 residents)	95 (13.2)	17 (8.0)	82 (11.9)	62 (10.9)	28 (11.7)
Medium (51–100 residents)	369 (51.3)	111 (52.4)	337 (52.5)	304 (53.6)	128 (53.3)
Large (> 101 residents)	255 (35.5)	84 (39.6)	228 (35.6)	201 (35.4)	84 (35.0)
Dementia-focused care (yes), *n* (%)	158 (22.0)	61 (28.8)	145 (22.4)	123 (21.7)	55 (22.9)
Availability of psychosocial services, *n* (%)					
Yes	156 (21.7)	42 (19.8)	131 (20.2)	106 (18.7)	44 (18.3)
No	563 (78.3)	170 (80.2)	516 (79.2)	461 (81.3)	196 (81.7)
Participatory leadership at care home, *n* (%)					
Yes	235 (32.7)	48 (22.6)	191 (29.5)	152 (26.8)	52 (21.7)
No	484 (67.3)	164 (77.4)	456 (70.5)	415 (73.2)	188 (78.3)
Access to professional development opportunities, *n* (%)					
Yes	631 (87.8)	176 (83.0)	563 (87.0)	488 (86.1)	195 (81.3)
No	88 (12.2)	36 (17.0)	84 (13.0)	79 (13.9)	45 (18.8)
Job Satisfaction (COPSOQ), *M* (*SD*)	45.43 (19.92)	39.13 (19.20)	43.25 (19.01)	40.58 (18.04)	34.76 (17.02)
Health and Posttraumatic Growth					
General health (COPSOQ), *M (SD*)	5.15 (2.23)	4.41 (2.09)	4.85 (2.09)	4.65 (2.03)	4.13 (1.96)
Self-reported COVID-19 infection history, *n* (%)					
Yes	176 (24.5)	56 (26.4)	150 (23.2)	121 (21.3)	48 (20.0)
No	543 (75.5)	156 (73.6)	497 (76.8)	446 (78.7)	192 (80.0)
Posttraumatic growth (PTGI), *M* (*SD*)	4.13 (2.30)	3.97 (2.18)	4.03 (2.22)	3.94 (2.19)	3.51 (2.19)
COVID-19-related impact on care homes					
COVID-19 cases among staff and/or residents (Yes), *n* (%)	719 (100.0)	212 (100.0)	647 (100.0)	567 (100.0)	240 (100.0)
COVID-19 related deaths among residents, *n* (%)					
Yes	549 (76.4)	179 (84.4)	494 (76.4)	442 (78.0)	189 (78.8)
No	170 (23.6)	33 (15.6)	153 (23.6)	125 (22.0)	51 (21.3)
Perceived staff absences during the second wave, *n* (%)					
Rather / strongly agree	658 (91.5)	204 (96.2)	600 (92.7)	536 (94.5)	229 (95.4)
Rather / strongly disagree	61 (8.5)	8 (3.8)	47 (7.3)	31 (5.5)	11 (4.6)
Satisfaction with quality of resident care and support, *n* (%)					
Very / rather satisfied	231 (32.1)	49 (23.1)	183 (28.3)	145 (25.6)	47 (19.6)
Very / rather dissatisfied	488 (67.9)	163 (76.9)	464 (71.7)	422 (74.4)	193 (80.4)
PTSD and Burnout					
PTSD (ITQ)					
Meeting PTSD screening criteria, *n* (%)	212 (29.5)	212 (100.0)	210 (32.5)	201 (35.4)	102 (42.5)
Personal burnout (CBI)					
* M* (*SD*)	70.64 (17.83)	79.38 (12.33)	74.76 (12.87)	76.59 (12.56)	80.42 (13.13)
Score ≥ 50, *n* (%)	647 (90.0)	210 (99.1)	647 (100.0)	560 (98.8)	239 (99.6)
Work-related burnout (CBI)					
* M* (*SD*)	64.18 (20.50)	74.58 (15.07)	67.97 (17.32)	72.29 (13.69)	77.74 (13.73)
Score ≥ 50, *n* (%)	567 (78.9)	201 (94.8)	560 (86.6)	567 (100.0)	235 (97.9)
Resident-related burnout (CBI)					
* M* (*SD*)	37.83 (23.01)	45.64 (24.85)	40.18 (22.58)	42.96 (22.13)	64.22 (12.93)
Score ≥ 50, *n* (%)	240 (33.4)	102 (48.1)	239 (36.9)	235 (41.4)	240 (100.0)
PTSD, personal burnout, work-related burnout, and/or resident-related burnout, *n* (%)	657 (91.4)				

Notes. *M* = Mean; *SD* = Standard deviation; PTSD = posttraumatic stress disorder; CBI = Copenhagen Burnout Inventory; PTGI = Posttraumatic Growth Inventory; COPSOQ = Copenhagen Psychosocial Questionnaire; ITQ = International Trauma Questionnaire. ^a^ “Other” employment includes marginal employment and unspecified types

### Relationship between PTSD, burnout and characteristics of participants and affiliated care homes

Higher PTSD levels were associated with female gender compared to male gender (*p* < .01, *r* = .10), absence of participatory leadership compared to its presence (*p* < .001, *r* = .17), no access to professional development opportunities compared to access (*p* < .05, *r* = .09), perceived staff absences during the second wave (rather/strongly agree vs. rather/strongly disagree; *p* < .001, *r* = .13), being very/rather dissatisfied with the quality of resident care and support compared to being very/rather satisfied (*p* < .001, *r* = .16), and COVID-19-related deaths among residents compared to no such deaths (*p* < .05, *r* = .09). Higher personal burnout was associated with female gender compared to male gender (*p* < .001, *r* = .14), no availability of psychosocial services compared to availability (*p* < .01, *r* = .11), absence of participatory leadership compared to its presence (*p* < .001, *r* = .27), no access to professional development opportunities compared to access (*p* < .001, *r* = .16), perceived staff absences (rather/strongly agree vs. rather/strongly disagree; *p* < .001, *r* = .13), being very/rather dissatisfied with the quality of resident care and support compared to being very/rather satisfied (*p* < .001, *r* = .27), and no history of COVID-19 infection compared to a history of infection (*p* < .01, *r* = .11). Higher work-related burnout was associated with female gender compared to male gender (*p* < .01, *r* = .11), no availability of psychosocial services compared to availability (*p* < .001, *r* = .13), absence of participatory leadership at care home compared to its presence (*p* < .001, *r* = .31), no access to professional development opportunities compared to access (*p* < .001, *r* = .15), perceived staff absences (rather/strongly agree vs. rather/strongly disagree; *p* < .001, *r* = .13), lower satisfaction with resident care and support (very/rather dissatisfied vs. very/rather satisfied; *p* < .001, *r* = .31), and no history of COVID-19 infection compared to a history of infection (*p* < .001, *r* = .13). Higher resident-related burnout was associated with part-time employment compared to full-time employment (*p* < .05, *r* = .09), absence of participatory leadership compared to its presence (*p* < .001, *r* = .23), no access to professional development opportunities compared to access (*p* < .001, *r* = .14), perceived staff absences (rather/strongly agree vs. rather/strongly disagree; *p* < .001, *r* = .15), lower satisfaction with resident care and support (very/rather dissatisfied vs. very/rather satisfied; *p* < .001, *r* = .25), and no history of COVID-19 infection compared to a history of infection (*p* < .01, *r* = .10) (see Table 2).

**Table 2:** Relationship between psychological burden and characteristics of participants

	PTSD		Personal burnout		Work-related burnout		Resident-related burnout	
	*Mdn* (*IQR*)	*U*	*Mdn* (*IQR*)	*U*	*Mdn* (*IQR*)	*U*	*Mdn *(*IQR*)	*U*
Characteristics of participants and affiliated care homes								
Gender								
Female (*n* = 628)	**8.0 (9.0)**	23679.0 **	**75.0 (16.67)**	21882.5***	**67.86 (25.0)**	23175.5**	37.5 (33.33)	28315.5
Male (*n* = 89)	**6.0 (8.0)**		**66.67 (29.17)**		**57.14 (25.71)**		33.33 (33.33)	
Employment status								
Full-Time (*n* = 484)	7.0 (10.0)	53775.5	75.0 (16.67)	56340.0	67.86 (28.57)	56640.5	**33.33 (29.17)**	50577.0*
Part-Time (*n* = 227)	8.0 (9.0)		70.83 (16.67)		67.86 (28.57)		**37.5 (33.3)**	
Size of care home								
Large (> 101 residents; *n* = 225)	8.0 (10.0)	54592.5	75.0 (20.83)	55859.0	67.86 (28.57)	57278.0	37.5 (33.33)	58958.5
Small (1–50 residents) / Medium (51–100 residents), (*n* = 464)	7.0 (9.0)		70.83 (16.67)		64.29 (28.57)		33.33 (33.33)	
Availability of psychosocial services								
Yes (*n* = 156)	7.0 (9.0)	40780.0	**70.83 (23.96)**	37456.5**	**60.71 (32.14)**	35786.0***	33.33 (33.33)	40275.0
No (*n* = 563)	8.0 (9.0)		**75.0 (16.67)**		**67.86 (25.0)**		37.5 (33.33)	
Participatory leadership at care home								
Yes (*n* = 235)	**6.0 (9.0)**	45296.0***	**66.67 (20.83)**	38007.5***	**57.14 (32.14)**	40998.0***	**29.17 (33.33)**	35029.5***
No (*n* = 484)	**8.0 (10.0)**		**75.0 (16.67)**		**71.43 (25.0)**		**41.67 (33.33)**	
Access to professional development opportunities								
Yes (*n* = 631)	**7.0 (9.0)**	23490.0*	**70.83 (16.67)**	20177.0***	**64.29 (28.57)**	20430.5***	**33.33 (29.17)**	20906.0***
No (*n* = 88)	**9.5 (9.75)**		**75.0 (16.67)**		**75.0 (25.0)**		**50.0 (41.67)**	
Health and Posttraumatic Growth								
Self-reported COVID-19 infection history								
Yes (*n* = 176)	7.0 (11.0)	43439.0	**70.83 (16.67)**	40653.0**	**62.5 (32.14)**	39428.0***	**29.17 (33.33)**	41153.0**
No (*n* = 543)	8.0 (9.0)		**75.0 (20.83)**		**67.86 (28.57)**		**37.5 (33.33)**	
COVID-19-related impact on care homes								
Perceived staff absences during the second wave								
Rather / strongly agree (*n* = 658)	**8.0 (9.0)**	14537.0 ***	**75.0 (16.67)**	14811.0***	**50.0 (41.07)**	13199.0***	**37.5 (33.33)**	13912.5***
Rather / strongly disagree (*n* = 170)	**5.0 (9.0)**		**66.67 (22.92)**		**67.86 (25.0)**		**25.0 (31.25)**	
COVID-19-related deaths among residents								
Yes (*n* = 549)	**8.0 (9.0)**	40716.5*	70.83 (16.67)	46627.5	67.86 (25.0)	46626.0	37.5 (33.33)	46523.0
No (*n* = 170)	**7.0 (8.0)**		75.0 (20.83)		67.86 (35.71)		37.5 (33.33)	
Satisfaction with quality of resident care and support								
Very / rather satisfied (*n* = 231)	**6.0 (9.0)**	45111.5***	**66.67 (25.0)**	37465.5***	**57.14 (32.14)**	34693.5***	**25.0 (29.17)**	38677.5***
Very / rather dissatisfied (*n* = 488)	**8.0 (10.0)**		**75.0 (16.67)**		**71.43 (25.0)**		**41.67 (33.33)**	

Notes. Group differences were tested using Mann–Whitney U tests for binary variables. Values reported in the test statistic columns represent *U* (Mann–Whitney U). *Mdn* = Median,* IQR* = interquartile range; *p < .05. **p < .01. ***p < .001. Significant results are also in bold

### Multivariate associations between PTSD, burnout and characteristics of participants and affiliated care homes

For PTSD, the multiple linear regression model explained a modest proportion of variance (adjusted *R²* = 0.144). PTSD was significantly associated with poorer general health (*β* = − 0.238, *p* < .001), lower job satisfaction (*β* = − 0.122, *p* < .01), COVID-19-related deaths among residents in care home (*β* = 0.104, *p* < .01), dementia-focused care (*β* = 0.102, *p* < .01) and female gender (*β* = 0.070, *p* < .05). For personal burnout, the regression model explained a substantial proportion of variance (adjusted *R²* = 0.501). Personal burnout was significantly associated with poorer general health (*β* = − 0.463, *p* < .001), lower job satisfaction (*β* = − 0.243, *p* < .001), female gender (*β* = 0.137, *p* < .001), absence of participatory leadership at care home (*β* = − 0.077, *p* < .01), younger age (*β* = − 0.073, *p* < .01), no COVID-19 infection history (*β* = − 0.068, *p* < .01), and lower posttraumatic growth (*β* = − 0.069, *p* < .05). For work-related burnout, the model explained a substantial proportion of variance (adjusted *R²* = 0.556). Work-related burnout was significantly associated with lower job satisfaction (*β* = − 0.415, *p* < .001), poorer general health (*β* = − 0.352, *p* < .001), lower posttraumatic growth (*β* = − 0.093, *p* < .001), female gender (*β* = 0.116, *p* < .001), no COVID-19 infection history (*β* = − 0.077, *p* < .001), absence of participatory leadership at care home (*β* = − 0.070, *p* < .05), and full-time employment (*β* = 0.038, *p* < .05). For resident-related burnout, the regression model explained a moderate proportion of variance (adjusted *R²* = 0.266). Resident-related burnout was significantly associated with lower job satisfaction (*β* = − 0.302, *p* < .001), poorer general health (*β* = − 0.195, *p* < .001), lower posttraumatic growth (*β* = − 0.099, *p* < .01), and no COVID-19 infection history (*β* = − 0.077, *p* < .05) (see Table 3).

**Table 3:** Multiple linear Regression analysis on PTSD (ITQ), Personal Burnout (CBI), Work-related Burnout (CBI) and Resident-related Burnout (CBI)

	PTSD		Personal Burnout		Work-related Burnout		Resident-related Burnout	
	*B* [*95% CI*]	*β*	*B* [*95% CI*]	*β*	*B* [*95*% *CI*]	*β*	*B* [*95% CI*]	*β*
(Intercept)	10.445*** **[7.41**,** 13.48]**		100.884*** **[93.91**,** 107.86]**		90.634*** **[83.07**,** 98.20]**		69.965*** **[59.05**,** 80.88]**	
Characteristics of participants and affiliated care homes								
Gender (Female)	1.246* **[0.02**,** 2.47]**	0.070*	7.354*** **[4.54**,** 10.17]**	0.137***	7.132*** **[4.08**,** 10.18]**	0.116***	− 0.217 [-4.62, 4.19]	− 0.003
Age	0.036 [-0.01, 0.08]	0.059	− 0.135** **[-0.23**,** − 0.04]**	− 0.073**	0.022 [-0.08, 0.13]	− 0.010	− 0.014 [-0.19, 0.11]	− 0.031
Employment status (Full-time)	− 0.146 [-1.03, 0.73]	− 0.012	0.718 [-1.30, 2.74]	0.020	2.319* **[0.13**,** 4.51]**	0.038*	-2.116 [-5.28, 1.04]	− 0.043
Size of care home (Large)	0.046 [-0.82, 0.91]	0.004	− 0.710 [0.48, -2.70]	0.019	-1.127 [-3.29, 1.04]	− 0.026	-1.818 [-4.94, 1.30]	− 0.038
Dementia-focused care (Yes)	1.454** **[0.47; 2.45]**	0.102**	1.671 [-0.61, 3.95]	0.039	0.507 [-1.96, 2.98]	0.010	− 0.383 [-3.94, 3.18]	− 0.007
Availability of psychosocial services (Yes)	− 0.032 [-1.05, 0.99]	− 0.002	− 0.797 [-3.14, 1.55]	− 0.018	− 0.821 [-3.36, 1.72]	− 0.017	2.347 [-1.32, 6.01]	0.042
Participatory leadership at care home (Yes)	− 0.661 [-1.63, 0.30]	− 0.052	-2.912** **[-5.13**,** − 0.70]**	− 0.077**	-3.035* **[-5.44**,** 0.63**]	− 0.070*	-2.102 [-5.57, 1.37]	− 0.043
Access to professional development opportunities (Yes)	− 0.625 [-1.91, 0.66]	− 0.035	− 0.646 [-3.60, 2.31]	− 0.012	1.348 [-1.86, 4.55]	0.022	-2.630 [-7.25, 1.99]	− 0.037
Job Satisfaction	− 0.036** **[-0.06**,** − 0.01]**	− 0.122**	− 0.218*** **[-0.28**,** − 0.16]**	− 0.243***	− 0.427*** **[-0.49**,** − 0.36]**	− 0.415***	− .349*** **[-0.44**,** − 0.26]**	− 0.302***
Health and Posttraumatic Growth								
General health	− 0.633*** **[-0.84**,** − 0.43]**	− 0.238***	-3.702*** **[-4.17**,** -3.23]**	− 0.463***	-3.236*** **[-3.75**,** -2.73]**	− 0.352***	-2.018*** **[-2.75**,** -1.28]**	− 0.195***
Self-reported COVID-19 infection history (Yes)	− 0.581 [-1.54, 0.38]	− 0.042	-2.810** **[-5.01**,** − 0.61]**	− 0.068 **	-4.352*** **[-6.74**,** -1.97]**	− 0.091***	-4.122* **[-7.56**,** − 0.68]**	− 0.077*
Posttraumatic Growth	0.053 [-0.13, 0.24]	0.021	− 0.538** **[-0.96**,** − 0.11]**	− 0.069**	− 0.829*** **[-1.29**,** − 0.37]**	− 0.093***	− 0.992** **[-1.66**,** − 0.33]**	− 0.099**
COVID-19-related impact on care homes								
Perceived staff absences during the second wave (Rather / strongly agree)	0.773 [-0.72, 2.27]	0.360	− 0.034 [-3.47, 3.40]	− 0.001	2.832 [-0.90, 6.56]	0.039	2.707 [-2.67, 8.09]	0.033
COVID-19-related deaths among residents (Yes)	1.449** **[0.47**,** 2.43]**	0.104**	2.113 [-0.15, 4.37]	0.050	2.291 [-16, 4.74]	0.048	0.930 [-2.61, 4.47]	0.017
Satisfaction with quality of resident care and support (Very / rather satisfied)	− 0.439 [-1.38, 0.51]	− 0.035	-2.081 [-4.25, 0.09]	− 0.055	-2.215 [-4.57, 0.14]	− 0.051	-3.071 [-6.47, 0.32]	− 0.062
*R*^*2*^ (adjusted *R*^*2*^)	0.162 (0.144)		0. 512 (0.501)		0.565 (0.556)		0.282 (0.266)	
*F* (*df1*, *df2*)	**9.072 (15**, 703) ***		**49.131 (15**, 703) ***		**60.790 (15**, 703) ***		**18.373 (15**, 703) ***	

*Notes. N =* 719. Model = “Inclusion” method in SPSS Statistics; *B *= unstandardized regression coefficient. *95%* *CI *= 95% confidence interval for *B*; The CI was rounded to the second decimal place; *β *= standardized regression coefficient; For dichotomous variables, regression coefficients represent comparisons between the category listed in parentheses (coded as 1) and the corresponding reference category (coded as 0). The dichotomous variables were coded as follows: Gender: Female (1) vs. Male/Other (0); Employment status: Full-time (1) vs. Part-time/Other (0); Size of care home: Large (1) vs. Small/Medium (0); Dementia-focused care: Yes (1) vs. No (0); Availability of psychosocial services: Yes (1) vs. No (0); Participatory leadership at care home: Yes (1) vs. No (0); Access to professional development opportunities: Yes (1) vs. No (0); Self-reported COVID-19 infection history: Yes (1) vs. No (0); Perceived staff absences during the second wave: Rather/strongly agree (1) vs. Rather/strongly disagree (0); COVID-19-related deaths among residents: Yes (1) vs. No (0); Satisfaction with quality of resident care and support: Very/rather satisfied (1) vs. Very/rather dissatisfied (0).*p < .05. **p < .01. ***p < .001. Significant results are also in bold

## Discussion

This study investigated PTSD and burnout among nurses in German care homes during the fourth and early fifth wave of the COVID-19 pandemic, examined associated factors and the availability of workplace resources. Using self-report measures, the vast majority (91.4%) of participants fulfilled the PTSD screening criteria and/or exceeded the indicative CBI cut-off for at least one dimension of burnout, indicating a high psychological burden within the present study sample. PTSD screening criteria were fulfilled by 29.5% of participants, which is in line with previous findings (29.1% pooled prevalence [8]), whereas a substantially larger proportion of participants exceeded the indicative cut-off for burnout (90.0% personal burnout in our study vs. 59.5% pooled prevalence in literature during the pandemic [7]). These discrepancies may be attributable to sample characteristics, contextual vulnerabilities, or timing of data collection during later pandemic phases, although interpretation of these findings over time remains inherently limited by the absence of comparable pre-pandemic data from German care homes. The voluntary online convenience sampling strategy may also have contributed to the high observed prevalence estimates through self-selection of nurses experiencing greater psychological burden. Notably, the high psychological burden observed in this sample coincided with limited access to workplace resources. Only one-third of participants reported the presence of participatory leadership at their care homes, and even fewer reported the availability of psychosocial services. These findings are consistent with earlier studies highlighting structural resource gaps and limited support in care homes [23].

Beyond prevalence estimates and resource availability, the multivariable analyses provide further insight into which factors are most relevant from a management perspective. Multivariate analyses revealed a range of variables associated with burnout and PTSD. Not all statistically significant associations were equally relevant in practical terms. Job satisfaction, general health, participatory leadership, and posttraumatic growth showed the most consistent and meaningful associations across burnout dimensions, whereas factors such as full-time employment were associated with comparatively small effect sizes. Personal burnout, work-related burnout and resident-related burnout exhibited overlapping associated factors, including lower job satisfaction, poorer general health, not having a COVID-19 infection history and lower posttraumatic growth. A finding contrary to previous research [16], was the association between no COVID-19 infection history and higher burnout. This may reflect prolonged frontline exposure without quarantine breaks, compounded by chronic fear of personal infection and infecting vulnerable residents, and limited opportunities for recovery among nurses who continued working throughout the pandemic (COR theory [31]). Alternative explanations, including healthy-worker or survivorship effects, cannot be excluded and warrant further investigation in longitudinal studies incorporating information on sick leave, quarantine periods, or work absences. Beyond shared risk factors, the burnout dimensions also showed distinct patterns of association, highlighting dimension-specific vulnerability profiles. Personal burnout was additionally associated with female gender, younger age and absence of participatory leadership at the care home. Work-related burnout showed similar associations, including full-time employment alongside female gender and absence of participatory leadership at the care home. Notably, given its higher prevalence, personal burnout represents a particularly pervasive form of psychological strain in this sample. Resident-related burnout was less prevalent and showed fewer significant associations, limited to those factors shared across all burnout dimensions. This suggests that, although interactions with residents may be emotionally demanding [25], they were not the factors most consistently associated with burnout symptoms in the present sample. Rather, the findings indicate that broader organizational and workplace-related factors were more consistently linked to burnout than resident-related demands in the present sample. Notably, the consistent association between the absence of participatory leadership at the care home and both personal and work-related burnout highlights participatory leadership as a potentially important organizational resource. This finding aligns with prior research demonstrating the protective function of participatory structures in healthcare settings [27, 28]. This interpretation is also consistent with the JD-R model, which emphasizes that burnout primarily arises when chronic job demands exceed available organizational resources. Accordingly, the observed associations suggest that organizational resources, including participatory leadership, may represent important considerations alongside individual-level approaches when developing strategies to address burnout in long-term care settings.

Although PTSD shared several associated factors with the burnout dimensions, including female gender, absence of participatory leadership, lower job satisfaction and poorer general health, it also demonstrated a distinct pattern of associations. In contrast to burnout, PTSD was additionally associated with resident-related factors, including dementia-focused care and COVID-19-related deaths among residents. These PTSD-specific associations may be understood in light of the distinctive emotional demands associated with long-term care nursing. Nurses in care homes often develop deep, quasi-familial bonds with residents, fostering strong emotional investment in their care [25]. Under pandemic conditions, marked by disproportionately high resident mortality, sudden clinical deterioration, strict infection control and social isolation measures [4–6], nurses were confronted with repeated deaths of residents, which, in combination with close emotional bonds, contributed to cumulative grief, feelings of helplessness, and moral distress. At the same time, nurses were required to continue providing care despite having limited to no resources or opportunities for emotional processing or recovery [25, 45]. Similarly, caring for residents with dementia under restricted activities and visitation bans, placed nurses in situations of sustained moral tension, further exacerbated by impaired communication and residents’ limited understanding of restrictions or the sudden absence of family members [46]. Taken together, these circumstances may help contextualize the observed associations between dementia-focused care, resident deaths, and PTSD, as they align with established trauma-exposure pathways, including repeated loss, moral injury, and insufficient recovery opportunities [47].

The present study provides several practical implications for nursing management in long-term care settings. Expanding access to structured psychosocial support, counselling, supervision, peer-support services, and protected time for reflective practice may help reduce burnout and psychological distress among nurses. The findings further highlight the importance of participatory leadership and formal mechanisms that enable nurses to actively contribute to organisational decision-making processes. Regular monitoring of staff well-being, psychological strain, and job satisfaction may facilitate the early identification of at-risk employees and support timely interventions. Particular attention should be paid to nurses working in dementia care and those exposed to COVID-19-related resident deaths, as these groups may be especially vulnerable to psychological burden. Structured debriefing following resident deaths, targeted support programmes for dementia-care teams, and trauma-informed leadership approaches may help strengthen resilience, improve staff retention, and support the delivery of high-quality care in long-term care settings.

### Strengths and limitations

To our knowledge, this is the first study focusing on burnout and PTSD among nurses working in care homes across Germany following the first waves of the pandemic. Key strengths include the large sample size (*N* = 719), enabling robust prevalence estimates across PTSD and the three burnout dimensions. The use of well-validated instruments (ITQ, CBI) ensures reliable measurement and facilitates international comparability. The differentiation of burnout into personal, work-related, and resident-related dimensions provides nuanced insights into distinct vulnerability profiles and specific risk/protective factors. However, the cross-sectional design does not allow for causal inference; sustained longitudinal panel studies monitoring workforce health are needed to establish temporal relationships and to estimate the impact of future events on health and well-being. Furthermore, as all key variables were assessed using self-report questionnaires at a single time point, common method variance may have contributed to some of the observed associations, particularly between conceptually related constructs such as job satisfaction, self-rated health, and burnout. Consequently, these associations should be interpreted with appropriate caution. Self-reported data introduces risks of social desirability and recall bias, potentially confounded by intervening life events. Measurement limitations include the use of four-point Likert scales, without neutral response options and single dichotomized items, particularly for the assessment of workplace and organizational factors (e.g. participatory leadership, psychosocial services, and perceived staff absences during second wave). These measurement characteristics may have reduced sensitivity and limited the construct validity of the respective measures. Similarly, although the single-item measure of self-rated general health derived from the COPSOQ is widely established and validated, a more comprehensive health measure may have provided a more detailed assessment and reduced measurement error. Moreover, the ICD-11-based ITQ differs from the common DSM-5 framework, potentially underrepresenting hyperarousal symptoms. Furthermore, participants completed the ITQ with reference to their experiences during the COVID-19 pandemic and work-related stressful events, which may not necessarily have constituted an ICD-11 qualifying traumatic event for all participants. Consequently, some degree of PTSD misclassification cannot be excluded. In addition, no standardized trauma exposure screener or assessment of previous traumatic experiences was administered prior to the ITQ. Consequently, we cannot exclude that pre-existing trauma exposure or PTSD vulnerability may have influenced participants’ responses. Accordingly, the reported PTSD prevalence and PTSD-specific associations should be interpreted with appropriate caution. Future studies should consider administering a standardized trauma exposure screener prior to the ITQ to allow for a more comprehensive assessment of trauma exposure prior to PTSD symptom assessment. Further, multiple univariate statistical tests were conducted to examine associations between several characteristics and different dimensions of burnout and PTSD. Although this approach allows for a detailed exploration of potential risk and protective factors, it increases the risk of Type I errors due to multiple testing. The univariate Mann-Whitney U analyses were conducted as exploratory analyses and should be considered secondary to the multivariable regression models, which constituted the primary analytical approach of this study. Therefore, no formal correction for multiple testing was applied. Consequently, statistically significant findings from the univariate analyses should be interpreted with caution and regarded as hypothesis-generating, as they may partly reflect chance associations. Furthermore, although data collection extended from the fourth into the early fifth wave of the COVID-19 pandemic, temporal differences in epidemiological conditions and workplace circumstances were not explicitly modelled. Further, the sample was recruited exclusively online, primarily via social media, which may have excluded nurses with limited internet access and introduced sampling bias. Although participants from all 16 German federal states were represented in the sample, the use of an online convenience sampling strategy limits the representativeness of the findings. In addition, due to the online recruitment strategy, it was not possible to establish a sampling frame, determine the number of invited care homes, or calculate an institutional response rate. No information identifying individual care homes was collected to ensure participant anonymity. Consequently, potential clustering effects at the facility level could not be examined. Future studies could consider collecting anonymized facility-level identifiers to enable multilevel analyses or other analytical approaches that account for potential within-facility clustering. Future studies could also incorporate additional occupational and individual-level variables, such as objective indicators of workload, staffing levels, shift patterns, and overtime, as well as information on prior mental health history and the use of psychological services, to provide a more comprehensive assessment of occupational demands and potential confounding. Moreover, the exceptionally high proportion of participants exceeding the indicative CBI cut-off for personal and work-related burnout should be interpreted with caution. As participation was voluntary and recruitment occurred primarily through online channels, self-selection bias cannot be excluded. Nurses experiencing greater psychological strain may have been more motivated to participate in a study focusing on mental health during the pandemic, potentially inflating the proportion of participants exceeding the indicative burnout cut-off. Therefore, these findings should not be interpreted as estimates of clinical burnout prevalence among all nurses working in German care homes. Lastly, overrepresentation of certain demographic groups may bias group comparisons and affect how gender effects are interpreted.

### Conclusion

Nurses working in German care homes, who participated in this voluntary online convenience sample, experienced substantial psychological burden during the COVID-19 pandemic, with a high proportion meeting PTSD screening criteria and/or exceeding the indicative CBI cut-off across the personal, work-related, and resident-related burnout dimensions. Although the non-probabilistic sampling strategy limits the generalizability of the prevalence figures to all nurses working in German care homes, the results provide valuable insights into a substantial psychological strain observed in this population and highlight factors associated with psychological burden that may be relevant for many care home settings. The study further found that workplace resources were limited, particularly with regard to participatory leadership and availability of psychosocial support. These resource constraints were associated with greater psychological burden in the present study. Vulnerable groups included female nurses, nurses of younger age, full-time employees, and work-involvement in dementia care or exposed to COVID-19-related resident deaths. Protective factors encompassed better general health, higher job satisfaction, and posttraumatic growth, highlighting the potential relevance of individual resilience in mitigating psychological strain. These findings are consistent with theoretical frameworks such as the JD-R model and COR theory, both of which emphasize the importance of balancing occupational demands and available resources in maintaining psychological well-being. These models propose that high job demands combined with limited resources may deplete psychological capacity, thereby increasing vulnerability to burnout and PTSD. Importantly, factors such as full-time employment, limited participatory leadership at care home, and caring for residents with dementia or those who died from or with COVID-19 were associated with higher levels of psychological burden and may be consistent with resource-related challenges described by the JD-R and COR frameworks. This may provide a coherent explanation for the elevated prevalence of psychological burden observed in this sample. These findings support consideration of organizational measures, including participatory leadership, psychosocial support, and workload management, as potentially relevant approaches to supporting nurses’ mental health and quality of care. Taken together, this study highlights the critical role of both individual and structural resources in mitigating psychological strain among care home staff.

## Supplementary Information

Below is the link to the electronic supplementary material.

Supplementary Material 1

Supplementary Material 2

## Data Availability

The dataset is available from the corresponding author on reasonable request.

## Abbreviations

CBI: Copenhagen burnout inventory
COR: Conservation of resources
COPSOQ: Copenhagen psychosocial questionnaire
ITQ: International trauma questionnaire
IQR: Interquartile range
JD-R: Job demands-resources
M: Mean
M-W: Mann-whitney U test
Mdn: Median
PTSD: Posttraumatic stress disorder
PTGI: Posttraumatic growth inventory
r: Effect size
RED: Cap research electronic data capture
SD: Standard deviation
VIF: Variance inflation factor
