# Supplementary material for: Burnout and posttraumatic stress among nurses in German care homes: a cross-sectional survey during the COVID-19 pandemic
Source: BMC Nurs. 2026 Jul 21;25:648. doi: 10.1186/s12912-026-05092-y (PMC13386803; doi:10.1186/s12912-026-05092-y)
Supplement: Supplementary file 1 — Supplementary Material 1 [file 12912_2026_5092_MOESM1_ESM.docx]

**Supplementary Methods**

**Questionnaire of:**

**Burnout and Posttraumatic Stress among Nurses in German Care Homes:
A Cross-Sectional Survey during the COVID-19 Pandemic**

(original and English translation)

**Angaben zur Person**

**01. Welches Geschlecht haben Sie?**

| Weiblich |  |  |  |  | ❑ |
| --- | --- | --- | --- | --- | --- |
| männlich |  |  |  |  | ❑ |
| divers |  |  |  |  | ❑ |

**02. Wie alt sind Sie?**

| 16 |  |  |  |  | ❑ |
| --- | --- | --- | --- | --- | --- |
| 17 |  |  |  |  | ❑ |
| 18 |  |  |  |  | ❑ |
| 19 |  |  |  |  | ❑ |
| 20 |  |  |  |  | ❑ |
| 21 |  |  |  |  | ❑ |
| 22 |  |  |  |  | ❑ |
| 23 |  |  |  |  | ❑ |
| 24 |  |  |  |  | ❑ |
| 25 |  |  |  |  | ❑ |
| 26 |  |  |  |  | ❑ |
| 27 |  |  |  |  | ❑ |
| 28 |  |  |  |  | ❑ |
| 29 |  |  |  |  | ❑ |
| 20 |  |  |  |  | ❑ |
| 21 |  |  |  |  | ❑ |
| 22 |  |  |  |  | ❑ |
| 23 |  |  |  |  | ❑ |
| 24 |  |  |  |  | ❑ |
| 25 |  |  |  |  | ❑ |
| 26 |  |  |  |  | ❑ |
| 27 |  |  |  |  | ❑ |
| 28 |  |  |  |  | ❑ |
| 29 |  |  |  |  | ❑ |
| 30 |  |  |  |  | ❑ |
| 31 |  |  |  |  | ❑ |
| 32 |  |  |  |  | ❑ |
| 33 |  |  |  |  | ❑ |
| 34 |  |  |  |  | ❑ |
| 35 |  |  |  |  | ❑ |
| 36 |  |  |  |  | ❑ |
| 37 |  |  |  |  | ❑ |
| 38 |  |  |  |  | ❑ |
| 39 |  |  |  |  | ❑ |
| 40 |  |  |  |  | ❑ |
| 41 |  |  |  |  | ❑ |
| 42 |  |  |  |  | ❑ |
| 43 |  |  |  |  | ❑ |
| 44 |  |  |  |  | ❑ |
| 45 |  |  |  |  | ❑ |
| 46 |  |  |  |  | ❑ |
| 47 |  |  |  |  | ❑ |
| 48 |  |  |  |  | ❑ |
| 49 |  |  |  |  | ❑ |
| 50 |  |  |  |  | ❑ |
| 51 |  |  |  |  | ❑ |
| 52 |  |  |  |  | ❑ |
| 53 |  |  |  |  | ❑ |
| 54 |  |  |  |  | ❑ |
| 55 |  |  |  |  | ❑ |
| 56 |  |  |  |  | ❑ |
| 57 |  |  |  |  | ❑ |
| 58 |  |  |  |  | ❑ |
| 59 |  |  |  |  | ❑ |
| 60 |  |  |  |  | ❑ |
| 61 |  |  |  |  | ❑ |
| 62 |  |  |  |  | ❑ |
| 63 |  |  |  |  | ❑ |
| 64 |  |  |  |  | ❑ |
| 65 |  |  |  |  | ❑ |
| 66 |  |  |  |  | ❑ |
| 67 |  |  |  |  | ❑ |

**03. In welcher Anstellungsart befinden Sie sich?**

| Vollzeit |  |  |  |  | ❑ |
| --- | --- | --- | --- | --- | --- |
| Teilzeit |  |  |  |  | ❑ |
| Geringfügige Beschäftigung |  |  |  |  | ❑ |
| Sonstige: ________ |  |  |  |  | ❑ |

**04. Wenn Sie Ihre Arbeitssituation insgesamt betrachten, wie zufrieden sind Sie mit…**

|  | sehr zufrieden | zufrieden | Teils Teils | Unzufrieden | Sehr unzufrieden |
| --- | --- | --- | --- | --- | --- |
| Ihre Berufsperspektiven | ❑ | ❑ | ❑ | ❑ | ❑ |
| Den Leuten mit denen Sie arbeiten? | ❑ | ❑ | ❑ | ❑ | ❑ |
| Den körperlichen Arbeitsbedingungen? | ❑ | ❑ | ❑ | ❑ | ❑ |
| Der Art und Weise wie Ihre Fähigkeiten genutzt werden? | ❑ | ❑ | ❑ | ❑ | ❑ |
| Der Art und Weise, wie Ihre Einrichtung geführt wird? | ❑ | ❑ | ❑ | ❑ | ❑ |
| Ihrem Lohn/Gehalt? | ❑ | ❑ | ❑ | ❑ | ❑ |
| Ihrer Arbeit insgesamt, unter Berücksichtigung aller Umstände? | ❑ | ❑ | ❑ | ❑ | ❑ |

**Angaben zu den Einrichtungen**

**05. Wie viele Pflegebedürftige leben aktuell in Ihrer Einrichtung**

| 1-50 |  |  |  |  | ❑ |
| --- | --- | --- | --- | --- | --- |
| 51-100 |  |  |  |  | ❑ |
| mehr als 100 |  |  |  |  | ❑ |

**06. Bietet Ihre Einrichtung den Mitarbeiter:innen in der Pflege psychosoziale Angebote (Supervision, psychologische Beratung etc.)**

| ja |  |  |  |  | ❑ |
| --- | --- | --- | --- | --- | --- |
| nein |  |  |  |  | ❑ |

**07. Bietet Ihre Einrichtung den Mitarbeiter:innen in der Pflege die Möglichkeit sich aktiv an Entscheidungsprozessen zu beteiligen?**

| ja |  |  |  |  | ❑ |
| --- | --- | --- | --- | --- | --- |
| nein |  |  |  |  | ❑ |

**08. Bietet Ihre Einrichtung den Mitarbeiter:innen in der Pflege die Möglichkeit Weiterbildungen zu besuchen?**

| ja |  |  |  |  | ❑ |
| --- | --- | --- | --- | --- | --- |
| nein |  |  |  |  | ❑ |

**09. Hat Ihre Einrichtung einen im Versorgungsvertrag vereinbarten pflegefachlichen Schwerpunkt?**

| ja |  |  |  |  | ❑ |
| --- | --- | --- | --- | --- | --- |
| nein |  |  |  |  | ❑ |

Welche(n) vereinbarte(n) pflegefachliche(n) Schwerpunkte(e) hat Ihre Einrichtung?

Mehrfachnennungen möglich

| Demenz |  |  |  |  | ❑ |
| --- | --- | --- | --- | --- | --- |
| Wachkoma |  |  |  |  | ❑ |
| Beatmung |  |  |  |  | ❑ |
| Gerontopsychiatrie |  |  |  |  | ❑ |
| Multiple Sklerose |  |  |  |  | ❑ |
| Psychische Erkrankungen |  |  |  |  | ❑ |
| Geistige Behinderungen |  |  |  |  | ❑ |
| Körperliche Behinderungen |  |  |  |  | ❑ |
| Palliativ Care/Sterbebegleitung |  |  |  |  | ❑ |
| Sonstiger Schwerpunkt: _______ |  |  |  |  | ❑ |

**Gesundheit und posttraumatisches Wachstum**

**10. Wenn Sie den besten denkbaren Gesundheitszustand mit 10 Punkten bewerten und den schlechtesten denkbaren Gesundheitszustand mit 0 Punkten:**

**Wie viele Punkte vergeben Sie dann für Ihren derzeitigen Gesundheitszustand?**

| 0 | 1 | 2 | 3 | 4 | 5 | 6 | 7 | 8 | 9 | 10 |
| --- | --- | --- | --- | --- | --- | --- | --- | --- | --- | --- |
| ❑ | ❑ | ❑ | ❑ | ❑ | ❑ | ❑ | ❑ | ❑ | ❑ | ❑ |

0 = schlechtester denkbarer Gesundheitszustand

10 = bester denkbarer Gesundheitszustand

**11. Als Nächstes würden wir gern von Ihnen erfahren, inwieweit die folgenden Aussagen auf Sie zutreffen.**

|  | Immer | Oft | Manch-mal | Selten | Nie/Fast nie |
| --- | --- | --- | --- | --- | --- |
| Wie häufig fühlen Sie sich müde? | ❑ | ❑ | ❑ | ❑ | ❑ |
| Wie häufig sind Sie körperlich erschöpft? | ❑ | ❑ | ❑ | ❑ | ❑ |
| Wie häufig sind Sie emotional erschöpft? | ❑ | ❑ | ❑ | ❑ | ❑ |
| Wie häufig denken Sie „Ich kann es nicht mehr ertragen“? | ❑ | ❑ | ❑ | ❑ | ❑ |
| Wie häufig fühlen Sie sich ausgelaugt? | ❑ | ❑ | ❑ | ❑ | ❑ |
| Wie häufig fühlen Sie sich schwach und krankheitsanfällig? | ❑ | ❑ | ❑ | ❑ | ❑ |
| Fühlen Sie sich nach einem Arbeitstag völlig fertig? | ❑ | ❑ | ❑ | ❑ | ❑ |
| Sind Sie morgens schon erschöpft, wenn Sie an den vor Ihnen liegenden Arbeitstag denken? | ❑ | ❑ | ❑ | ❑ | ❑ |
| Haben Sie das Gefühl, dass Sie jede Arbeitsstunde ermüdet? | ❑ | ❑ | ❑ | ❑ | ❑ |
| Haben Sie genug Energie für Familie und Freunde während Ihrer Freizeit? | ❑ | ❑ | ❑ | ❑ | ❑ |

|  | sehr stark | stark | etwas | wenig | sehr wenig, gar nicht |
| --- | --- | --- | --- | --- | --- |
| Belastet Sie Ihre Arbeit emotional? | ❑ | ❑ | ❑ | ❑ | ❑ |
| Frustriert Sie Ihre Arbeit? | ❑ | ❑ | ❑ | ❑ | ❑ |
| Fühlen Sie sich aufgrund Ihrer Arbeit ausgebrannt? | ❑ | ❑ | ❑ | ❑ | ❑ |
| Belastet Sie die Arbeit mit den Bewohner:innen | ❑ | ❑ | ❑ | ❑ | ❑ |
| Raubt Ihnen die Arbeit mit den Bewohner:innen Ihre Energie? | ❑ | ❑ | ❑ | ❑ | ❑ |
| Frustriert Sie die Arbeit mit den Bewohner:innen? | ❑ | ❑ | ❑ | ❑ | ❑ |
| Haben Sie das Gefühl, dass Sie in der Arbeit mit den Bewohner:innen mehr geben als Sie bekommen? | ❑ | ❑ | ❑ | ❑ | ❑ |

|  | Immer | Oft | Manch-mal | Selten | Nie/Fast nie |
| --- | --- | --- | --- | --- | --- |
| Haben Sie die Arbeit mit Bewohner:innen satt? | ❑ | ❑ | ❑ | ❑ | ❑ |
| Fragen Sie sich manchmal, wie lange Sie die Arbeit mit Bewohner:innen noch durchhalten können? | ❑ | ❑ | ❑ | ❑ | ❑ |

**12. Haben Sie sich im Verlauf der Pandemie nachweislich mit dem Coronavirus infiziert?**

| ja |  |  |  |  | ❑ |
| --- | --- | --- | --- | --- | --- |
| nein |  |  |  |  | ❑ |

**13. Im Folgenden sind Probleme und Beschwerden aufgelistet, die bei Menschen als Folgen von traumatischen oder belastenden Lebenserfahrungen auftreten.**

**Bitte denken Sie bei der Beantwortung der nachfolgenden Fragen an die Corona-Pandemie und mögliche belastende Erlebnisse auf der Arbeit die damit zusammenhingen.**

**Bitte lesen Sie jede Aussage gründlich durch und bewerten dann, wie sehr dieses Problem im letzten Monat belastet hat.**

|  | Gar nicht | Ein bisschen | mittelmäßig | ziemlich | Sehr stark |
| --- | --- | --- | --- | --- | --- |
| Aufwühlende Träume, in denen Teile Covid-19-bezogener Erlebnisse wieder passierten oder in einen klaren Bezug zu einem Covid-19-bezogenen Erlebnis hatten. | ❑ | ❑ | ❑ | ❑ | ❑ |
| Intensive Bilder oder Erinnerungen haben, die manchmal auftauchen und bei denen Sie sich fühlen, als ob das Covid-19-bezogene Erlebnis jetzt und hier gerade noch einmal stattfindet. | ❑ | ❑ | ❑ | ❑ | ❑ |
| Vermeiden, dass etwas von Innen an das Covid-19-bezogene Erlebnis erinnert (zum Beispiel Gedanken, Gefühle oder Körperempfindungen). | ❑ | ❑ | ❑ | ❑ | ❑ |
| Vermeiden, dass etwas von außen an das Covid-19-bezogene Erlebnis erinnert (zum Beispiel Menschen, Orte, Gespräche, Dinge, Tätigkeiten oder Situationen). | ❑ | ❑ | ❑ | ❑ | ❑ |
| Extrem wachsam sein, aufmerksam oder „auf der Hut“ sein. | ❑ | ❑ | ❑ | ❑ | ❑ |
| Sich kribbelig fühlen oder leicht erschreckbar sein | ❑ | ❑ | ❑ | ❑ | ❑ |
| Haben sich die oben genannten Probleme im letzten Monat… |  |  |  |  |  |
| …auf ihre Beziehungen zu anderen Menschen oder ihre soziale Kontakte ausgewirkt? | ❑ | ❑ | ❑ | ❑ | ❑ |
| …auf Ihre Arbeit oder Ihre Arbeitsfähigkeit ausgewirkt? | ❑ | ❑ | ❑ | ❑ | ❑ |
| …auf irgendeinen anderen wichtigen Lebensbereich ausgewirkt, wie zum Beispiel Kindererziehung, Leistungen für Schule oder Universität oder sonstige wichtige Aktivitäten? | ❑ | ❑ | ❑ | ❑ | ❑ |

**14. Neben den Belastungen, die die Corona-Pandemie mit sich gebracht hat, hat sie aber auch Anregungen für Veränderungen geschafft. Darüber hinaus bieten Krisen auch die Möglichkeit an Ihnen zu wachsen.**

**Mit den folgenden Fragen soll erfasst werden, ob sich aufgrund der Corona-Krise bestimmt Dinge in Ihrem Leben verändert haben. Klicken Sie bitte Zutreffendes an.**

|  | Überhaupt nicht | etwas | Sehr stark |
| --- | --- | --- | --- |
| Ich entwickelte ein Gefühl des Selbstvertrauens. | ❑ | ❑ | ❑ |
| Ich weiß jetzt, dass ich mit Schwierigkeiten umgehen kann. | ❑ | ❑ | ❑ |
| Ich bin mehr in der Lage, die Wendungen zu akzeptieren, die Dinge nehmen. | ❑ | ❑ | ❑ |
| Ich entdeckte, dass ich stärker bin, als ich dachte. | ❑ | ❑ | ❑ |

**Auswirkungen von COVID-19 auf die Einrichtungen**

**15. Gab es seit Beginn der Pandemie (01.03.2020) nachgewiesene Covid-19-Infektionen unter Ihren Kolleg:innen in der Pflege?**

| ja |  |  |  |  | ❑ |
| --- | --- | --- | --- | --- | --- |
| nein |  |  |  |  | ❑ |

**16. Gab es seit Beginn der Pandemie (01.03.2020) nachgewiesene Covid-19-Infektionen unter den von Ihnen versorgten Bewohnerinnen?**

| ja |  |  |  |  | ❑ |
| --- | --- | --- | --- | --- | --- |
| nein |  |  |  |  | ❑ |

Sind seit Beginn der Pandemie (01.03.2020) Bewohnerinnen in Ihrer Einrichtung mit oder an einer Covid-19-Infektion verstorben?

| ja |  |  |  |  | ❑ |
| --- | --- | --- | --- | --- | --- |
| nein |  |  |  |  | ❑ |

**17. Darüber hinaus, kam es während der zweiten Infektionswelle (01. Oktober 2020 bis 31. Januar 2021) zu Personenausfällen?**

| stimmt überhaupt nicht zu |  |  |  |  | ❑ |
| --- | --- | --- | --- | --- | --- |
| stimmt eher nicht zu |  |  |  |  | ❑ |
| stimmt eher zu |  |  |  |  | ❑ |
| stimmt voll und ganz zu |  |  |  |  | ❑ |

**18. Wie beurteilen Sie insgesamt die Qualität der Pflege und Betreuung für die Bewohner:innen Ihrer Einrichtung unter den Gegebenheiten der Corona-Pandemie?**

| Sehr unzufrieden |  |  |  |  | ❑ |
| --- | --- | --- | --- | --- | --- |
| Eher unzufrieden |  |  |  |  | ❑ |
| Eher zufrieden |  |  |  |  | ❑ |
| Sehr zufrieden |  |  |  |  | ❑ |

**Supplementary Methods**

**Questionnaire of:**

**Burnout and Posttraumatic Stress among Nurses in German Care Homes:
A Cross-Sectional Survey during the COVID-19 Pandemic**

(original and English translation)

English Translation:

**Characteristics of participants**

**01. What is your gender?**

| female |  |  |  |  | ❑ |
| --- | --- | --- | --- | --- | --- |
| male |  |  |  |  | ❑ |
| diverse |  |  |  |  | ❑ |

**02. How old are you?**

| 16 |  |  |  |  | ❑ |
| --- | --- | --- | --- | --- | --- |
| 17 |  |  |  |  | ❑ |
| 18 |  |  |  |  | ❑ |
| 19 |  |  |  |  | ❑ |
| 20 |  |  |  |  | ❑ |
| 21 |  |  |  |  | ❑ |
| 22 |  |  |  |  | ❑ |
| 23 |  |  |  |  | ❑ |
| 24 |  |  |  |  | ❑ |
| 25 |  |  |  |  | ❑ |
| 26 |  |  |  |  | ❑ |
| 27 |  |  |  |  | ❑ |
| 28 |  |  |  |  | ❑ |
| 29 |  |  |  |  | ❑ |
| 20 |  |  |  |  | ❑ |
| 21 |  |  |  |  | ❑ |
| 22 |  |  |  |  | ❑ |
| 23 |  |  |  |  | ❑ |
| 24 |  |  |  |  | ❑ |
| 25 |  |  |  |  | ❑ |
| 26 |  |  |  |  | ❑ |
| 27 |  |  |  |  | ❑ |
| 28 |  |  |  |  | ❑ |
| 29 |  |  |  |  | ❑ |
| 30 |  |  |  |  | ❑ |
| 31 |  |  |  |  | ❑ |
| 32 |  |  |  |  | ❑ |
| 33 |  |  |  |  | ❑ |
| 34 |  |  |  |  | ❑ |
| 35 |  |  |  |  | ❑ |
| 36 |  |  |  |  | ❑ |
| 37 |  |  |  |  | ❑ |
| 38 |  |  |  |  | ❑ |
| 39 |  |  |  |  | ❑ |
| 40 |  |  |  |  | ❑ |
| 41 |  |  |  |  | ❑ |
| 42 |  |  |  |  | ❑ |
| 43 |  |  |  |  | ❑ |
| 44 |  |  |  |  | ❑ |
| 45 |  |  |  |  | ❑ |
| 46 |  |  |  |  | ❑ |
| 47 |  |  |  |  | ❑ |
| 48 |  |  |  |  | ❑ |
| 49 |  |  |  |  | ❑ |
| 50 |  |  |  |  | ❑ |
| 51 |  |  |  |  | ❑ |
| 52 |  |  |  |  | ❑ |
| 53 |  |  |  |  | ❑ |
| 54 |  |  |  |  | ❑ |
| 55 |  |  |  |  | ❑ |
| 56 |  |  |  |  | ❑ |
| 57 |  |  |  |  | ❑ |
| 58 |  |  |  |  | ❑ |
| 59 |  |  |  |  | ❑ |
| 60 |  |  |  |  | ❑ |
| 61 |  |  |  |  | ❑ |
| 62 |  |  |  |  | ❑ |
| 63 |  |  |  |  | ❑ |
| 64 |  |  |  |  | ❑ |
| 65 |  |  |  |  | ❑ |
| 66 |  |  |  |  | ❑ |
| 67 |  |  |  |  | ❑ |

**03. What is your employment status?**

| Full-time |  |  |  |  | ❑ |
| --- | --- | --- | --- | --- | --- |
| Part-time |  |  |  |  | ❑ |
| Minor employment |  |  |  |  | ❑ |
| other: ________ |  |  |  |  | ❑ |

**04. Regarding your work in general. How pleased are you with**

|  | very satisfied | satisfied | neither/ nor | unsatisfied | highly unsatisfied |
| --- | --- | --- | --- | --- | --- |
| …your work prospects? | ❑ | ❑ | ❑ | ❑ | ❑ |
| …the people you work with? | ❑ | ❑ | ❑ | ❑ | ❑ |
| …the physical working conditions? | ❑ | ❑ | ❑ | ❑ | ❑ |
| …the way your group is run? | ❑ | ❑ | ❑ | ❑ | ❑ |
| …the way your abilities are used? | ❑ | ❑ | ❑ | ❑ | ❑ |
| …your salary? | ❑ | ❑ | ❑ | ❑ | ❑ |
| …your job as a whole, everything taken into consideration? | ❑ | ❑ | ❑ | ❑ | ❑ |

**Characteristics of affiliated care homes**

**05. How many residents currently live in your facility?**

| 1-50 |  |  |  |  | ❑ |
| --- | --- | --- | --- | --- | --- |
| 51-100 |  |  |  |  | ❑ |
| larger than 100 |  |  |  |  | ❑ |

**06. Does your institution offer psychosocial services (such as supervision, psychological counseling, etc.)?**

| yes |  |  |  |  | ❑ |
| --- | --- | --- | --- | --- | --- |
| no |  |  |  |  | ❑ |

**07. Does your institution provide nursing staff with the opportunity to actively participate in decision-making processes?**

| yes |  |  |  |  | ❑ |
| --- | --- | --- | --- | --- | --- |
| no |  |  |  |  | ❑ |

**08. Does your institution allow nursing staff to attend further training or continuing education courses?**

| yes |  |  |  |  | ❑ |
| --- | --- | --- | --- | --- | --- |
| no |  |  |  |  | ❑ |

**09. Does your facility have a specialized nursing focus as agreed upon in the care contract?**

| yes |  |  |  |  | ❑ |
| --- | --- | --- | --- | --- | --- |
| no |  |  |  |  | ❑ |

What are the agreed-upon nursing specialties at your facility?

Multiple selections possible

| Dementia |  |  |  |  | ❑ |
| --- | --- | --- | --- | --- | --- |
| Persistent vegetative state |  |  |  |  | ❑ |
| Ventilation |  |  |  |  | ❑ |
| Gerontopsychiatry |  |  |  |  | ❑ |
| Multiple sclerosis |  |  |  |  | ❑ |
| Mental Illness |  |  |  |  | ❑ |
| Intellectual Disabilities |  |  |  |  | ❑ |
| Physical Disabilites |  |  |  |  | ❑ |
| Palliativ Care/End-of-Life Care |  |  |  |  | ❑ |
| Other area of focus: _______ |  |  |  |  | ❑ |

**Health and posttraumatic growth**

**10. If you evaluate the best conceivable state of health at 10 points and the worst at 0 points: How many points do you then give to your present state of health? Please select the corresponding number.**

| 0 | 1 | 2 | 3 | 4 | 5 | 6 | 7 | 8 | 9 | 10 |
| --- | --- | --- | --- | --- | --- | --- | --- | --- | --- | --- |
| ❑ | ❑ | ❑ | ❑ | ❑ | ❑ | ❑ | ❑ | ❑ | ❑ | ❑ |

0 = worst conceivable state of health

10 = best conceivable state of health

**11. Next, we would like to find out to what extent the following statements apply to you.**

|  | always | often | sometimes | seldom | never/almost never |
| --- | --- | --- | --- | --- | --- |
| How often do you feel tired? | ❑ | ❑ | ❑ | ❑ | ❑ |
| How often are you physically exhausted? | ❑ | ❑ | ❑ | ❑ | ❑ |
| How often are you emotionally exhausted? | ❑ | ❑ | ❑ | ❑ | ❑ |
| How often do you think: “I can’t take it anymore”? | ❑ | ❑ | ❑ | ❑ | ❑ |
| How often do you feel worn out? | ❑ | ❑ | ❑ | ❑ | ❑ |
| How often do you feel weak and susceptible to illness? | ❑ | ❑ | ❑ | ❑ | ❑ |
| Do you feel worn out at the end of the working day? | ❑ | ❑ | ❑ | ❑ | ❑ |
| Are you exhausted in the morning at the thought of another day at work? | ❑ | ❑ | ❑ | ❑ | ❑ |
| Do you feel that every working hour is tiring for you? | ❑ | ❑ | ❑ | ❑ | ❑ |
| Do you have enough energy for family and friends during leisure time? (inverse scoring) | ❑ | ❑ | ❑ | ❑ | ❑ |

|  | to a very high degree | to a high degree | somewhat | to a low degree | to a very low degree |
| --- | --- | --- | --- | --- | --- |
| Is your work emotionally exhausting? | ❑ | ❑ | ❑ | ❑ | ❑ |
| Does your work frustrate you? | ❑ | ❑ | ❑ | ❑ | ❑ |
| Do you feel burnt out because of your work? | ❑ | ❑ | ❑ | ❑ | ❑ |
| Do you find it hard to work with residents? | ❑ | ❑ | ❑ | ❑ | ❑ |
| Does it drain your energy to work with residents? | ❑ | ❑ | ❑ | ❑ | ❑ |
| Do you find it frustrating to work with residents? | ❑ | ❑ | ❑ | ❑ | ❑ |
| Do you feel that you give more than you get back when you work with residents? | ❑ | ❑ | ❑ | ❑ | ❑ |

|  | always | often | sometimes | seldom | never/almost never |
| --- | --- | --- | --- | --- | --- |
| Are you tired of working with residents? | ❑ | ❑ | ❑ | ❑ | ❑ |
| Do you sometimes wonder how long you will be able to continue working with residents? | ❑ | ❑ | ❑ | ❑ | ❑ |

**12. Did you test positive for the coronavirus during the pandemic?**

| yes |  |  |  |  | ❑ |
| --- | --- | --- | --- | --- | --- |
| no |  |  |  |  | ❑ |

**13. Indicate for each of the statements below the degree to which this change occurred in your life as a result of the corona-crisis, using the following scale.**

0 = I did not experience this change as a result of my crisis.

1 = I experienced this change to a very small degree as a result of my crisis.

2 = I experienced this change to a small degree as a result of my crisis.

3 = I experienced this change to a moderate degree as a result of my crisis.

4 = I experienced this change to a great degree as a result of my crisis.

5 = I experienced this change to a very great degree as a result of my crisis.

|  | 1 | 2 | 3 | 4 | 5 |
| --- | --- | --- | --- | --- | --- |
| I have a greater feeling of self-reliance. | ❑ | ❑ | ❑ | ❑ | ❑ |
| I know better that I can handle difficulties. | ❑ | ❑ | ❑ | ❑ | ❑ |
| I am better able to accept the way things work out. | ❑ | ❑ | ❑ | ❑ | ❑ |
| I discovered that I'm stronger than I thought I was. | ❑ | ❑ | ❑ | ❑ | ❑ |

**14. Below are a number of problems that people sometimes report in response to traumatic or stressful life events. Please read each item carefully, then circle one of the numbers to the right to indicate how much you have been bothered by that problem in the past month.**

|  | not at all | a little bit | Moderately | Quite a bit | extremely |
| --- | --- | --- | --- | --- | --- |
| Having upsetting dreams that replay part of the experience or are clearly related to the experience? | ❑ | ❑ | ❑ | ❑ | ❑ |
| Having powerful images or memories that sometimes come into your mind in which you feel the experience is happening again in the here and now? | ❑ | ❑ | ❑ | ❑ | ❑ |
| Avoiding internal reminders of the experience (for example, thoughts, feelings, or physical sensations)? | ❑ | ❑ | ❑ | ❑ | ❑ |
| Avoiding external reminders of the experience (for example, people, places, conversations, objects, activities, or situations)? | ❑ | ❑ | ❑ | ❑ | ❑ |
| Being “super-alert”, watchful, or on guard? | ❑ | ❑ | ❑ | ❑ | ❑ |
| Feeling jumpy or easily startled? | ❑ | ❑ | ❑ | ❑ | ❑ |
| In the past month have the above problems: |  |  |  |  |  |
| Affected your relationships or social life? | ❑ | ❑ | ❑ | ❑ | ❑ |
| Affected your work or ability to work? | ❑ | ❑ | ❑ | ❑ | ❑ |
| Affected any other important part of your life such as parenting, or school or college work, or other important activities? | ❑ | ❑ | ❑ | ❑ | ❑ |

**COVID-19-related impact on care homes**

**15. Since the beginning of the pandemic (March 1, 2020), have there been confirmed COVID-19 infections among nursing staff?**

| yes |  |  |  |  | ❑ |
| --- | --- | --- | --- | --- | --- |
| No |  |  |  |  | ❑ |

**16. Since the beginning of the pandemic (March 1, 2020), have there been confirmed COVID-19 infections among the residents?**

| yes |  |  |  |  | ❑ |
| --- | --- | --- | --- | --- | --- |
| no |  |  |  |  | ❑ |

Since the beginning of the pandemic, have any residents in your facility died due to or in connection with a COVID-19 infection?

| yes |  |  |  |  | ❑ |
| --- | --- | --- | --- | --- | --- |
| no |  |  |  |  | ❑ |

**17. Did staff absences occur during the second infection wave (October 1, 2020 to January 31, 2021)?**

| strongly disagree |  |  |  |  | ❑ |
| --- | --- | --- | --- | --- | --- |
| rather disagree |  |  |  |  | ❑ |
| rather agree |  |  |  |  | ❑ |
| strongly agree |  |  |  |  | ❑ |

**18. How would you rate the overall quality of care and support for residents in your facility under the conditions of the COVID-19 pandemic?**

| very dissatisfied |  |  |  |  | ❑ |
| --- | --- | --- | --- | --- | --- |
| rather dissatisfied |  |  |  |  | ❑ |
| rather satisfied |  |  |  |  | ❑ |
| very satisfied |  |  |  |  | ❑ |
