# Supplementary material for: Burnout and posttraumatic stress among nurses in German care homes: a cross-sectional survey during the COVID-19 pandemic
Source: BMC Nurs. 2026 Jul 21;25:648. doi: 10.1186/s12912-026-05092-y (PMC13386803; doi:10.1186/s12912-026-05092-y)
Supplement: Supplementary file 2 — Supplementary Material 2 [file 12912_2026_5092_MOESM2_ESM.docx]

**Burnout and Posttraumatic Stress among Nurses in German Care Homes:
A Cross-Sectional Survey during the COVID-19 Pandemic**

*Supplementary Material*

**Supplementary Table S1.** Variance inflation factors (VIF) for the predictors included in the multiple linear regression models.

| *Variable* | *VIF* |
| --- | --- |
| Gender (Female) | 1.092 |
| Age | 1.045 |
| Employment status (Full-time) | 1.056 |
| Size of care home (Large) | 1.070 |
| Dementia-focused care (Yes) | 1.044 |
| Availability of psychosocial services (Yes) | 1.097 |
| Participatory leadership at care home (Yes) | 1.272 |
| Access to professional development opportunities (Yes) | 1.102 |
| Job Satisfaction | 1.618 |
| General health | 1.289 |
| Self-reported COVID-19 infection history (Yes) | 1.052 |
| Posttraumatic Growth | 1.114 |
| Perceived staff absences during the second wave (Rather / strongly agree) | 1.079 |
| COVID-19-related deaths among residents (Yes) | 1.086 |
| Satisfaction with quality of resident care and support (Very / rather satisfied) | 1.207 |
| ***Notes*.** VIF = variance inflation factor. Values are reported for all predictors included in the multiple linear regression models. All observed VIF values were below the commonly recommended threshold of 5, indicating no evidence of problematic multicollinearity. Dichotomous variables were coded as described in Table 3. | |

**Supplementary Table S2.** Pearson correlation matrix of the variables included in the multiple linear regression models.

| Variable | 1 | 2 | 3 | 4 | 5 | 6 | 7 | 8 | 9 | 10 | 11 | 12 | 13 | 14 | 15 | 16 | 17 | 18 | 19 |
| --- | --- | --- | --- | --- | --- | --- | --- | --- | --- | --- | --- | --- | --- | --- | --- | --- | --- | --- | --- |
| 1 | 1.00 |  |  |  |  |  |  |  |  |  |  |  |  |  |  |  |  |  |  |
| 2 |  | 1.00 |  |  |  |  |  |  |  |  |  |  |  |  |  |  |  |  |  |
| 3 |  |  | 1.00 |  |  |  |  |  |  |  |  |  |  |  |  |  |  |  |  |
| 4 |  |  |  | 1.00 |  |  |  |  |  |  |  |  |  |  |  |  |  |  |  |
| 5 | .094 | .162 | .129 | .009 | 1.00 |  |  |  |  |  |  |  |  |  |  |  |  |  |  |
| 6 | .047 | -.123 | -.041 | -.050 | .012 | 1.00 |  |  |  |  |  |  |  |  |  |  |  |  |  |
| 7 | -.038 | -.018 | -.014 | -.083 | -.078 | -.078 | 1.00 |  |  |  |  |  |  |  |  |  |  |  |  |
| 8 | .056 | .029 | .033 | .004 | -.033 | .108 | .039 | 1.00 |  |  |  |  |  |  |  |  |  |  |  |
| 9 | .119 | .052 | .027 | -.007 | .040 | .035 | .019 | .070 | 1.00 |  |  |  |  |  |  |  |  |  |  |
| 10 | -.048 | -.124 | -.143 | -.060 | -.033 | .026 | .065 | .118 | .047 | 1.00 |  |  |  |  |  |  |  |  |  |
| 11 | -.162 | -.282 | -.320 | -.231 | -.029 | -.058 | .138 | -.070 | .010 | .223 | 1.00 |  |  |  |  |  |  |  |  |
| 12 | -.099 | -.151 | -.144 | -.148 | -.053 | .073 | -.016 | .002 | .075 | .073 | .170 | 1.00 |  |  |  |  |  |  |  |
| 13 | -.269 | -.519 | -.637 | -.465 | .007 | .047 | .108 | -.047 | -.012 | .194 | .381 | .239 | 1.00 |  |  |  |  |  |  |
| 14 | -.310 | -.623 | -.585 | -.372 | -.059 | .082 | -.035 | -.063 | -.020 | .062 | .176 | .092 | .430 | 1.00 |  |  |  |  |  |
| 15 | -.058 | -.102 | -.127 | -.109 | .003 | .012 | .052 | -.003 | -.091 | -.017 | .079 | .005 | .059 | .036 | 1.00 |  |  |  |  |
| 16 | -.052 | -.230 | -.273 | -.230 | .064 | .046 | .038 | -.025 | .113 | .065 | .164 | .033 | .253 | .196 | -.014 | 1.00 |  |  |  |
| 17 | .122 | .143 | .195 | .137 | .049 | .029 | -.010 | .038 | -.019 | -.009 | -.043 | -.038 | -.177 | -.159 | -.036 | -.043 | 1.00 |  |  |
| 18 | .098 | .006 | .011 | -.003 | -.015 | .066 | -.032 | .139 | .019 | .047 | .011 | -.058 | -.001 | .046 | .157 | .018 | .136 | 1.00 |  |
| 19 | -.159 | -.290 | -.319 | -.252 | -.025 | .036 | .048 | -.074 | -.041 | .100 | .251 | .157 | .367 | .227 | .059 | .139 | -.133 | .018 | 1.00 |
| ***Notes*.** Pearson product-moment correlation coefficients. N = 719. Variables: (1) PTSD; (2) Personal Burnout; (3) Work-related Burnout; (4) Resident-related Burnout; (5) Gender (Female); (6) Age; (7) Employment status (Full-time); (8) Size of care home (Large); (9) Dementia-focused care (Yes); (10) Availability of psychosocial services (Yes); (11) Participatory leadership at care home (Yes); (12) Access to professional development opportunities (Yes); (13) Job Satisfaction; (14) General health; (15) Self-reported COVID-19 infection history (Yes); (16) Posttraumatic Growth; (17) Perceived staff absences during the second wave (Rather/strongly agree); (18) COVID-19-related deaths among residents (Yes); (19) Satisfaction with quality of resident care and support (Very/rather satisfied). Dichotomous variables were coded as described in Table 3. | | | | | | | | | | | | | | | | | | | |

**Supplementary Figure S3:** Distribution of participants across CBI score categories for the three Copenhagen Burnout Inventory (CBI) subscales.

**A. Absolute frequencies (*n*)**

Personal burnout (CBI) Work-related burnout (CBI) Resident-related burnout (CBI)

**B. Percentages (%)**

Personal burnout (CBI) Work-related burnout (CBI) Resident-related burnout (CBI)

*Notes.* Severity categories were based on established indicative Copenhagen Burnout Inventory (CBI) score ranges: <25 = “low”; 25 to <50 = “moderate”; 50 to <75 = “high”; and ≥75 = “very high”. Panels A and B present identical distributions expressed as absolute frequencies and percentages, respectively.
